# Bibliographical

**Published:** 1879-06

**Authors:** 


### BIBLIOGRAPHICAL.

ELEMENTS OF DENTAL MATERIA MEDICA and THERAPEUTICS with Pharmacopœia. Second edition. By James Stockton, L. D. S., N. C. S. England. Three hundred and nineteen pages.

This work comes nearer supplying a long recognized want in dental text books than anything heretofore published. It is an excellent book of reference for the dental practitioner. It contains a brief description of all the therapeutic agents and preparations that will usually be required by the dentist. They are arranged and classified and so described as to render their use understandable to any one of good professional attainments. It is not designed to take the place of more extensive works on the subject, but to be a *vade mecum*, a book of ready reference, and this niche it fills to an admirable degree.

It should be in the hands of every dentist; no one can afford to be without it.

It is published by Lindsay & Blakiston, of Philadelphia, and this is enough, as to the mechanical execution of the work. Any bookseller either has or can procure it.

